# Orthodontic digital workflow: devices and clinical applications

**DOI:** 10.1590/2177-6709.26.6.e21spe6

**Published:** 2021-12-15

**Authors:** Taís de Morais Alves da CUNHA, Inessa da Silva BARBOSA, Karolinne Kaila PALMA

**Affiliations:** 1 Instituto Prime de Ensino, Curso de Especialização em Ortodontia (Salvador/BA, Brazil).

**Keywords:** Dental bonding, Orthodontic brackets, Digital Orthodontics

## Abstract

**Introduction:**

The digital technology has contributed to improve and simplify diagnosis, treatment planning and execution in Orthodontics. Among CAD/CAM system (Computer-Aided Design / Computer-Aided Manufacturing) applications in Orthodontics, we highlight the installation and removal of fixed appliance, clear aligners, customized appliances, and retainers fabricated in digital environment. This approach has several advantages for practitioner and patient, as it enhances appliances precision, directly interferes in treatment time and predictability. Even with all the benefits arising from the digital workflow, few orthodontists have adopted this technique in their clinical practice, most due to high cost and lack of technical preparation for proper execution.

**Objectives:**

Thus, given the importance of digital technology to improve specialty performance and the still incipient incorporation of digital flow in Orthodontics, the purpose of this article is to describe the available resources and clinical applications of the CAD/CAM technology in Orthodontics.

## RESUMO

**Introdução:**

A utilização da tecnologia digital tem contribuído para aprimorar e facilitar o diagnóstico, planejamento e execução dos tratamentos ortodônticos. Entre as diversas aplicações do sistema CAD/CAM (*Computer-Aided Design / Computer-Aided Manufacturing*) destacamos a sequência clínica de tratamento ortodôntico com aparelho fixo, tratamentos com alinhadores estéticos, aparelhos personalizados sobre mini-implantes e confecção de contenções totalmente em ambiente digital. Essa abordagem apresenta diversas vantagens para o profissional e o paciente, na medida em que aumenta a precisão dos aparelhos, interfere diretamente no tempo de tratamento e na previsibilidade do resultado. Embora os benefícios decorrentes do fluxo de trabalho digital sejam evidentes, poucos ortodontistas adotaram essa técnica na prática clínica, principalmente devido ao custo elevado e à falta de preparo técnico para a adequada execução.

**Objetivo:**

Dessa forma, diante da importância da utilização da tecnologia digital para aprimorar a atuação ortodôntica e da ainda incipiente incorporação do fluxo digital na prática clínica, o objetivo do presente artigo é apresentar recursos disponíveis e aplicações clínicas da tecnologia CAD/CAM na Ortodontia.

## INTRODUCTION

From the description of the first orthodontic appliance in 1728 by Pierre Fauchard^1^ to the current wide use of aesthetic aligners, Orthodontics has undergone a great evolution of technique and materials. Some events may be highlighted such as the Edgewise appliance patented by Edward Angle,^2^ the direct bonding of orthodontic accessories to enamel^3^ and more recently the use of CAD/CAM (Computer-Aided Design / Computer-Aided Manufacturing) for diagnosis, treatment planning and customized orthodontic appliances fabrication.^4^

The CAD/CAM technology allows three-dimensional (3D) images manipulation through computer software and 3D printing of customized devices in different materials.^5^ Among the orthodontic applications, there are the precise and efficient aligners production, customized devices, indirect bonding trays, as well as the virtual brackets debonding for retainer manufacture.^6^

CAD/CAM technology is supported by three pillars: the digital image acquisition of patients’ dental arches; the visualization and manipulation of these images in specific software; and the files 3D-printing, whether the devices designed or the models in which the devices will be made. This process is called Digital Workflow.

The objective of applying this technology in Orthodontics is to reduce the professional’s chair and laboratory time, as well as turn the treatments faster, predictable, aesthetic and more comfortable to patients^5^. Even with all the benefits arising from the digital workflow, orthodontists still underuse technology in their practice, perhaps due to lack of technical knowledge to introduce it and its high cost. 

Considering the advent of CAD/CAM technology a distinct evolutionary milestone in Orthodontics history, since it provides great possibility of use in clinical practice, with potential benefits for patient and practitioner, the aim of this article is to describe the CAD/CAM system and the clinical applications of the digital workflow in Orthodontics. 

## DIGITAL FILES ACQUISITION

The substitution of traditional casting by intraoral scan represents a paradigm shift. The direct capture of the dental arches surface topography unveils a virtual universe of possibilities and benefits for clinical Orthodontics.

Conventional casting is operator-dependent and presents several sensitive steps that can contribute to decrease the cast’s accuracy. In addition, it is a procedure that can lead to patient’s anxiety and discomfort. Intraoral scanning, on the other hand, incurs fewer repetitions, shorter chair time, greater patient comfort and has the very high digital precision.^7^

It is important to highlight that intraoral scanner captures images by projection of a source of laser light or structured light, without interaction with biological tissues.^8^ The technology used by the sensor to obtain the image determines the speed, resolution, and accuracy of the scanner.^7^ ^,^ ^8^ These devices present specific software that process data and produce the 3D virtual image of dental arches.^7^ ^-^ ^9^

Incorporating intraoral scanning in clinical practice requires an initial investment in technology and knowledge.^10^ The equipment selection should consider elements such as the need for surface opacification, the speed and scan accuracy, the camera tip size and possibility of sterilization, and the ability to produce color images. Besides that, some systems are closed, therefore not allowing a free interface with CAD software, and may present additional upgrade costs.^6^ ^-^ ^10^

It is of utmost importance to consider the relevant features of the equipment, such as: open system, without upgrade cost, dimensions and necessity of a computer. However, it is not necessary to have a scanner in the office to work in digital flow, since this service can be outsourced.

## VIEWING, EDITING AND PRINTING DIGITAL FILES

**Figure 1:**
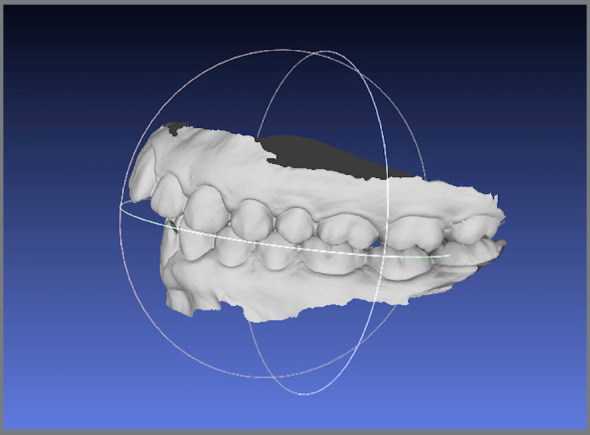
STL file of 3D digital model, in lateral view.

The data of dental arches surface topography correspond to a mesh of triangulated points and are usually saved on computer in a Standard Triangulation Language (STL) format file (Fig 1). Capture systems allow the user to export the data directly to the orthodontic lab or access the files in software with specific tools for 3D images manipulation.^5^ ^,^ ^7^

Virtual casts allow clinicians to rapidly obtain diagnosis information, such as: arch width and perimeter, model discrepancies, Bolton discrepancy, overjet and overbite. In addition, diagnostic setups may be performed and reviewed with patient, to discuss different treatment alternatives.^5^ ^,^ ^11^

Orthodontics specific software may be acquired, to allow using all its features, or charged on demand. In general, they are of easy manipulation, however, they require proper training for clinical practice incorporation.^12^

The next step of the digital workflow is 3D printing of captured or manipulated images. In Orthodontics, the additive manufacturing printing methods are more used, such as the stereolithography (SLA), which solidifies liquid resins with ultraviolet light; and the fused deposition modeling (FDM), which uses thermoplastic polymers filaments. The progress to in-office 3D printing is probably the next change in orthodontic clinic.^6^ ^,^ ^7^

## CAD/CAM TECHNOLOGY IN ORTHODONTICS

There are unlimited applications of the CAD/CAM system in Orthodontics. From virtual models it is possible to plan and manufacture devices for interceptive and corrective Orthodontics with conventional brackets or clear aligners.

Additionally, there is an improvement in the interdisciplinary treatment plan, due to the ability of different 3D technologies integration; in this way, the tomography images may be superimposed to STL file, enabling the analysis of root inclination, alveolar bone thickness and basal bone. Such integration provides better virtual treatment planning for orthognathic surgery, impacted tooth or extraction situations.^7^

### CLEAR ALIGNERS

The most common use of digital flow in Orthodontics is the digital setups and fabrication of clear aligners, although it is only part of its clinical applications. In the same way, setups allow greater predictability,^11^ irrespective to the orthodontic appliance used in the treatment. 

Traditionally, the orthodontic setup was carried out in the plaster model from the separation of the crowns and repositioning in wax. With digital technology treatment simulation process has become faster and more practical. The models referring to the treatment stages are automatically generated by the setup software and are used for the aligners production.^11^

It is important to note that, unlike real biological dental movements, virtual movements are unlimited and often the results may not be realistic.^13^ Therefore, an additional investment in knowledge of aligners biomechanics is recommended to clinicians, in order to understand the importance of attachments to increase anchorage and efficiency of movements, as well as the need to perform movements in stages, overcorrect or use auxiliary mechanics.

### AUXILIARY APPLIANCES IN DIGITAL LABORATORY

The manufacture of auxiliary appliances, especially those used in Interceptive Orthodontics, is a possibility of digital technology. This approach eliminates the clinical appointment of transfer molding, and the devices are made with laser welding, which is more biocompatible. Thus, it is important to place separation elastics before scanning, for banded devices.^6^ ^,^ ^14^

In addition, the technology allows the manufacture of auxiliary devices with dentoskeletal anchorage. For hybrid devices planning, the virtual model can be superimposed on the patient’s tomography, making it possible to produce guides for anchorage devices insertion by CAD/CAM, as well as to design customized rings for mini-implants.^15^

### DIGITAL WORKFLOW FOR CONVENTIONAL ORTHODONTIC TREATMENT

The digital workflow for bonding fixed orthodontic appliance allows greater precision in the brackets placement^16^ and reduces the laboratory time required for conventional indirect bonding.^17^ In addition, it eliminates the adhesion impairments in the resin/adhesive interface.^16^ ^-^ ^18^

Indirect bonding in Orthodontics arose to minimize isolation and view difficulties inherent to the conventional procedure.^19^ ^,^ ^20^ Besides that, correct brackets placement and enamel adhesion success are essentials for orthodontic treatment efficiency; in this sense, indirect bonding provides better accuracy in accessories positioning, and turns the bonding appointment faster and more comfortable for patients.^21^ ^,^ ^22^

### CAD/CAM TECHNOLOGY FOR ORTHODONTIC FIXED APPLIANCE ASSEMBLY

**Figure 2:**
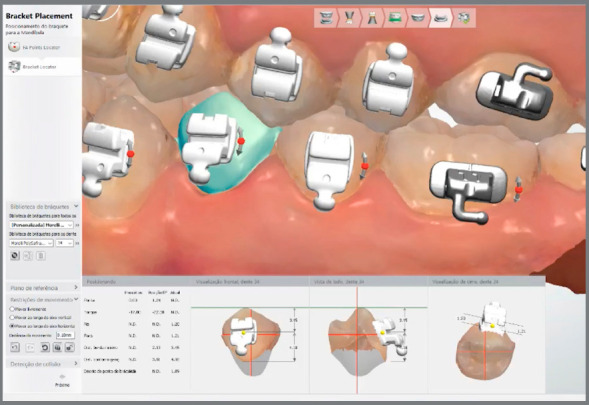
Virtual brackets placement in Ortho Analyzer^TM^ software.

The orthodontic appliance virtual assembly may be performed in the Ortho Analyzer^TM^ software (3Shape, Copenhagen, Denmark).^23^ The same bracket’s trademark to be used in patient must be selected in the software virtual bracket’s library (Fig 2). 

The accessory placement is guided by accurate digital measurements tools with screen view in multiple angles, free of saliva and soft tissues. Thus, it is possible to review the alignment with the chosen bracket position.^5^

**Figure 3:**
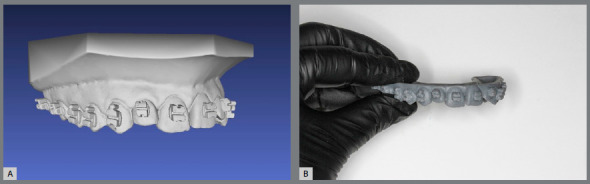
A) STL file of 3D model with virtually attached brackets; B) 3D printed model for transfer tray confection.

**Figure 4:**
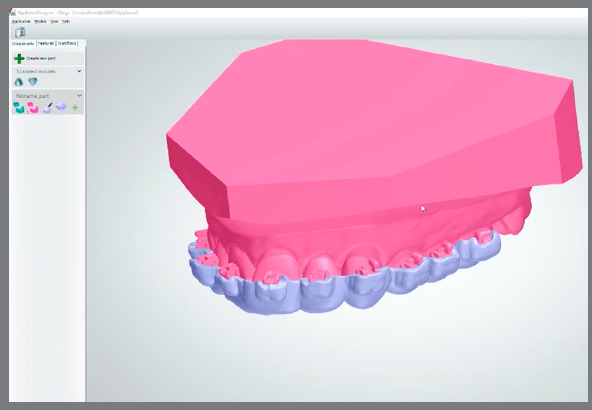
Virtual design of a transfer tray at the Appliance Designer^TM^ software.

Afterwards a new STL file is generated, with brackets attached to digital models, and may be sent for 3D printing (Fig 3) or used to virtually design a bonding tray in the Appliance Designer^TM^ software (3Shape, Copenhagen, Denmark) (Fig 4). 

Then, there are two principal pathways to produce the indirect bonding tray: pressure-formed and direct 3D-printed.

The STL archives of virtual models with brackets placed should be sent to an SLA (Stereolithography) or DLP (Digital Light Processing) printer.^6^ This printing technologies are preferable, since the model accuracy is very important to achieve tray precise fit to patient teeth. 

**Figure 5:**
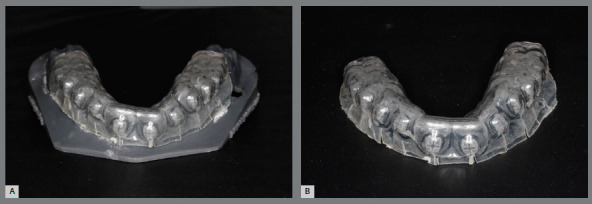
A) Indirect bonding guide fabricated on 3D-printed model, by the double pressure-forming technique (B).

The indirect bonding guide fabrication on the printed resin model with brackets (Fig 5) is carried out by the double pressure-forming technique.^19^ As previously described^19^ ^,^ ^24^ for conventional indirect bonding, the dual tray owns an inner flexible material covering brackets and teeth, and an outer rigid material to guide and stabilize. 

**Figure 6:**
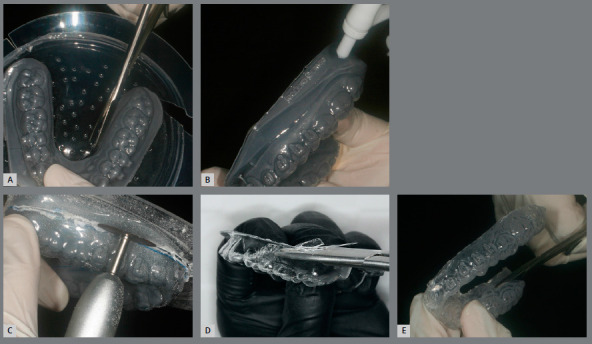
A) Pressure-formed silicone tray. B) Spray of separating medium. C) Cutout of the double plates from 3D-printed model. D) PET-G tray trim and E) silicone tray trim.

The laboratory procedure consists in thermoplasticization of a soft tray (silicon; Bio-Art, SP, Brazil) and a 1-mm thick tray (acetate; Cristal Bio-Art). It is important to perform previous isolation with lubrication spray (KAVO), before stamping the rigid tray over the soft one. The silicone tray is cut 3mm beyond gingival teeth margin and should undergo vertical cutouts to smooth removal after the orthodontic appliance bonding (Fig 6). 

**Figure 7:**
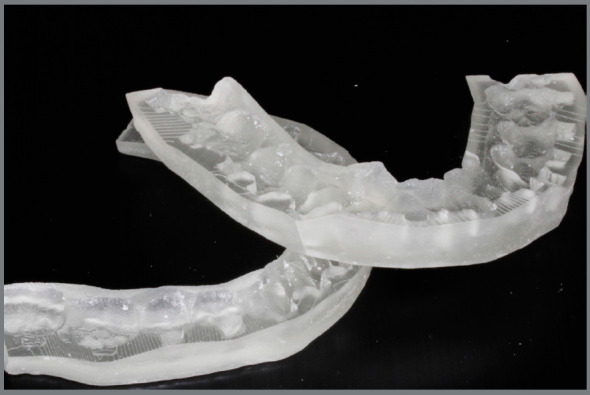
3D-printed transfer tray in clear flexible resin.

Another way to produce the indirect bonding guide is through direct printing the virtually designed customized tray^5^ ^,^ ^25^ on a clear, flexible and biocompatible (Yller, Rio Grande do Sul, Brazil) material, to allow the clinical tray removal without lifting brackets and the light-curing. This is the full digital workflow with computer design and manufacturing technique to fabricate the customized tray in very high precision (Fig 7). 

**Figure 8:**
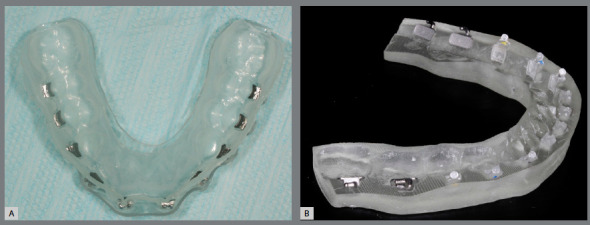
A) Brackets placed in pressure-formed bonding double tray. (B) Brackets placed on 3D-printed bonding tray.

Regardless of the tray fabrication method, in the digital indirect bonding the brackets can be positioned in their respective locations prior to the bonding session^5^ (Fig 8). Printed trays provide greater accuracy of bracket insertion locations^5^.

**Figure 9:**
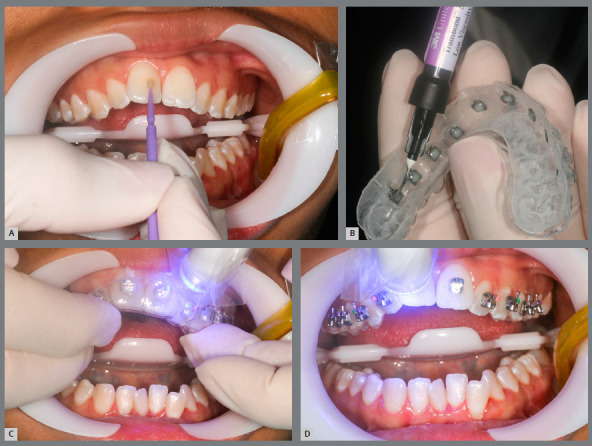
Clinical procedure: A) self-etch primer application; B) low viscosity resin application to brackets bases; C) light-curing, with transfer tray in place; D) light-curing after bonding tray removal.

The clinical procedure is simple, practical and efficient. Thus, prophylaxis with pumice, relative teeth isolation, acid etching and primer or self-etch primer application should be performed. Then, a thin layer of low viscosity resin (Transbond Supreme^TM^) should be applied to brackets base, then the transfer tray may be positioned in patient’s mouth (Fig 9). After removing excess adhesives, a first light-curing should be performed. Then, after tray removal, another light-curing may be executed to provide better adhesion. 

**Figure 10:**
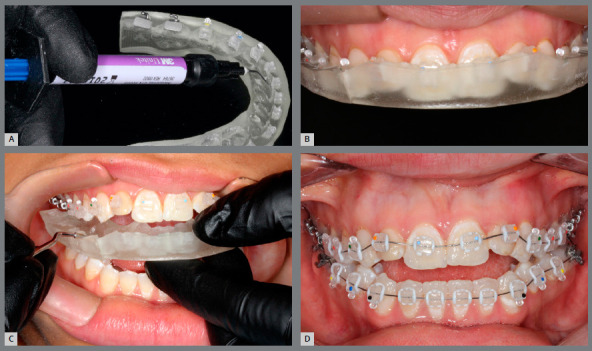
Clinical stages: A) low viscosity resin application to brackets placed on 3D-printed tray; B) 3D-printed bonding tray in place, after adhesive excess removal and light-curing; C) transfer tray removal; D) full orthodontic fixed appliance bonded in a single appointment.

The pressured-formed bonding tray removal should be more careful, and it is important to first remove the acetate plate, perform a new polymerization and then remove the silicone tray. When using the printed tray, the clinical stage is faster and with less risk of brackets debonding during removal (Fig 10). 

### CAD/CAM TECHNOLOGY FOR REMOVAL OF ORTHODONTIC FIXED APPLIANCE

**Figure 11:**
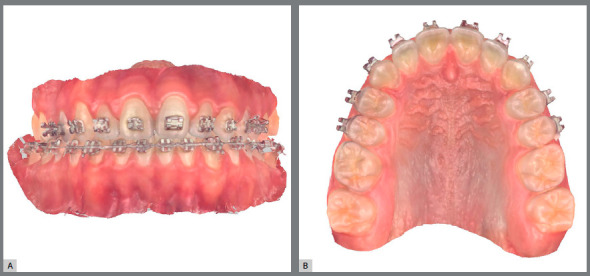
Intraoral scans at treatment finishing: A) virtual models in occlusion, frontal view; B) virtual maxillary arch model in occlusal view, without archwire.

For orthodontic fixed appliance removal in digital flow, it is necessary to perform an intraoral scan when the orthodontic treatment is finished. For this scan, it is important to remove the archwires and molars tubes (Fig 11).

**Figure 12:**
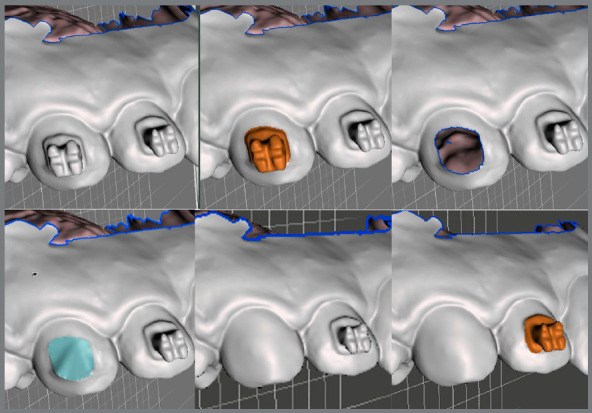
Virtual brackets removal on an STL manipulation software.

**Figure 13:**
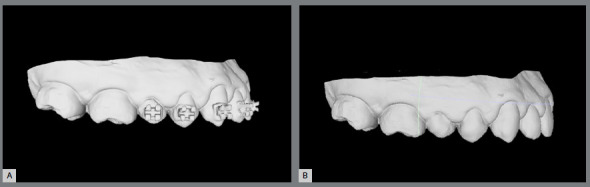
A) STL file of maxillary arch acquired at treatment finishing. B) STL file of maxillary arch after digital removal of accessories.

The brackets may be virtually removed in the Meshmixer (Autodesk Inc., USA) (Fig 12), and then a STL file of the virtual models without brackets is generated and may be exported to a 3D printer and used to produce an orthodontic retainer device^6^ (Fig 13).

**Figure 14:**
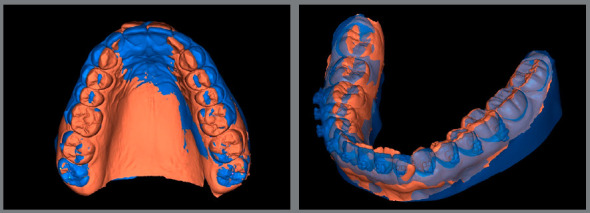
Superimposition of initial and final STL files, after brackets virtual removal.

Another great advantage of the digital models is that the final scan may be superimposed to the initial scan, to evaluate the tooth movement performed (Fig 14).

**Figure 15:**
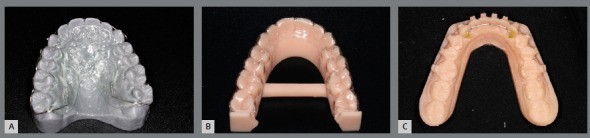
Orthodontic retainers fabricated in digital workflow: A) upper wraparound appliance, B) thermoformed retainer and C) bonded lingual retainer.

**Figure 16:**
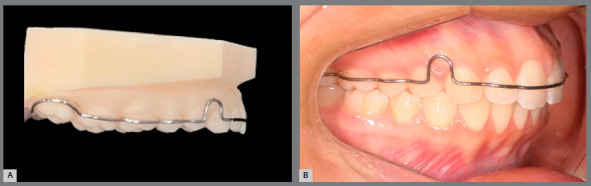
A) Orthodontic upper removable retainer, placed immediately after debonding (B), with precise adaptation.

The printed models allow the manufacturing of thermoformed, lingual-bonded or Hawley-type retainer (Fig 15), which can be placed with high precision immediately at the debonding appointment^6^ (Fig 16).

**Figure 17:**
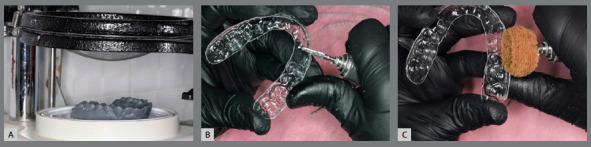
Digital workflow for manufacturing of thermoformed retainer appliance: A) thermoforming; B) finishing and C) polishing.

**Figure 18:**
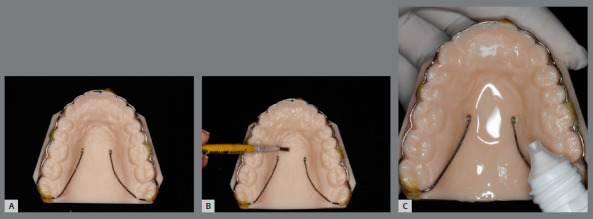
Digital workflow for acrylic removable upper retainer confection: A) hooks fixed to 3D-printed model, B) application of separating medium and (C) self-curing acrylic resin manipulation by additive method.

Due to excellent precision requirement of working models, the SLA and DLP printers are preferable. The appliance manufacturing does not differ from the protocol of conventional cast models, and is illustrated in Figures 17 and 18.

Digital workflow reduces the number of appointments to deliver the retainer appliance. Another advantage is that the same model can be used to produce replacement retainers in case of appliance loss or damage. Furthermore, if any relapse is noticed during the retention phase, it is possible to virtually correct teeth position and produce thermoformed appliance for realignment.^6^

**Figure 19:**
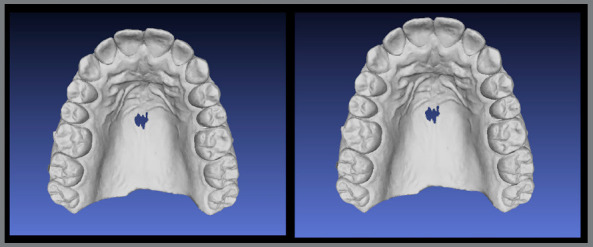
Virtual setup for orthodontic treatment finishing after brackets removal.

**Figure 20:**
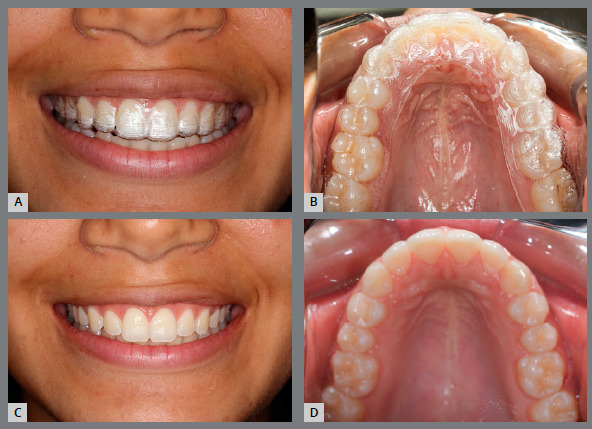
Thermoformed aligner used for finishing teeth movements.

These devices can be manufactured not only in cases of relapse, but also for refinement of orthodontic finishing. This approach may decrease the time of fixed appliance treatment and increase the predictability of final occlusal relationships (Figs 19 and 20). 

## DISCUSSION

Digital workflow for indirect bonding in Orthodontics features numerous advantages, including: lack of physical impressions, pouring gypsum cast and decreased laboratory working time.^16^ ^,^ ^17^ ^,^ ^26^

The correct brackets positioning is very important, since any error incorporated may reflect in significant dental position deviation, impairing orthodontic finishing. Thus, treatment time extends, as bracket repositioning or additional bends are necessary.^19^ ^-^ ^21^

The advent of digital indirect bonding enables greater precision and standardization in bracket positioning, due to digital measuring tools, improved view and manipulation of virtual models.^16^ ^,^ ^19^ Consequently, the digital bonding may reduce treatment duration and contribute to better occlusal relationships.^16^ ^,^ ^17^ ^,^ ^21^

Another remarkable benefit of this approach is the possibility of total appliance assembly in a unique bonding appointment, which may also contribute to decrease treatment duration.^27^

To the best of our knowledge, there are no studies comparing the precision of flexible printed trays and those thermoformed on printed models, yet the higher accuracy of printed models has been described^26^ and also the clinical feasibility of a rigid 3D-printed tray.^28^ The indirect bonding tray, prototyped in transparent and flexible material, probably promotes a better adjustment to the brackets and teeth, in addition to being more durable and can be sectioned to be used as a single bracket guide, in cases of debonding during treatment. 

The digital indirect bonding has been described as more accurate than direct bonding,^16^ however it is still necessary to access shear bond strength of accessories placed with printed guides. Brackets bases are not contaminated with resin or cast material, which may improve bond strength, compared to conventional indirect bonding techniques.^25^ ^,^ ^29^

El-Timamy et al.^25^ proposed the Cone Beam Computerized Tomography (CBCT) for virtual bracket placement, to allow visualization of root axis and enhance precision. It is also possible to superimpose CBCT and oral scans,^23^ with the aim of obtaining more accurate posttreatment root parallelism. However, CBCT ionizing radiation dose can interfere with biological tissues, thus requiring criteria for orthodontic indication.^30^ The intraoral scans images have the advantage of not producing any biological effect, and may be widely utilized.

Adopting CAD/CAM technology to virtual brackets removal enable to install the retainers in both arches on debonding appointment. Moreover, models printed in very high definition may lead to highest precision of retainer devices, thus minimizing problems with appliances adaptation. Besides that, it promotes comfort for orthodontist and patient, by eliminating impressions of dental arches with brackets.

There is an expectation of evolution towards the adoption of digital indirect bonding and debonding in clinical practice. Further scan superimpositions should be used to check tooth movement and review patient progress even with brackets. 

It is important to keep in mind that digital workflow is wider than aligners. Although the high cost is still an impairment, CAD/CAM system should be explored for conventional Orthodontics, as incorporating this technique may improve the clinical practice and make diagnosis and treatment planning easier, since it may reduce chair time and the number of appointments, enhance patient comfort, and may influence predictability of results and allow an environment of communication with patient and among professionals involved in the clinical case. 

## CONCLUSION

Digital indirect bonding and virtual bracket removal may contribute to decreased orthodontic treatment time, eliminate clinical and laboratory steps, and favoring greater patient comfort and better accuracy and predictability. However, the adoption of CAD/CAM technology in Orthodontics presents a higher financial cost and the need for professional training.
